# *Ex vivo* physiological compression of human osteoarthritis cartilage modulates cellular and matrix components

**DOI:** 10.1371/journal.pone.0222947

**Published:** 2019-09-24

**Authors:** Paolo Dolzani, Elisa Assirelli, Lia Pulsatelli, Riccardo Meliconi, Erminia Mariani, Simona Neri

**Affiliations:** 1 Laboratorio di Immunoreumatologia e Rigenerazione Tissutale, IRCCS Istituto Ortopedico Rizzoli, Bologna, Italy; 2 Unità di Medicina e Reumatologia, IRCCS Istituto Ortopedico Rizzoli, Bologna, Italy; 3 Dipartimento di Scienze Biomediche e Neuromotorie, Università di Bologna, Bologna, Italy; 4 Dipartimento di Scienze Mediche e Chirurgiche, Università di Bologna, Bologna, Italy; University of Patras, GREECE

## Abstract

Mechanical stimulation appears to play a key role in cartilage homeostasis maintenance, but it can also contribute to osteoarthritis (OA) pathogenesis. Accumulating evidence suggests that cartilage loading in the physiological range contributes to tissue integrity maintenance, whereas excessive or reduced loading have catabolic effects. However, how mechanical stimuli can regulate joint homeostasis is still not completely elucidated and few data are available on human cartilage. We aimed at investigating human OA cartilage response to *ex vivo* loading at physiological intensity. Cartilage explants from ten OA patients were subjected to *ex vivo* controlled compression, then recovered and used for gene and protein expression analysis of cartilage homeostasis markers. Compressed samples were compared to uncompressed ones in presence or without interleukin 1β (IL-1β) or interleukin 4 (IL-4). Cartilage explants compressed in combination with IL-4 treatment showed the best histological scores. Mechanical stimulation was able to significantly modify the expression of collagen type II (collagen 2), aggrecan, SOX9 transcription factor, cartilage oligomeric matrix protein (COMP), collagen degradation marker C2C and vascular endothelial growth factor (VEGF). Conversely, ADAMTS4 metallopeptidase, interleukin 4 receptor alpha (IL4Rα), chondroitin sulfate 846 epitope (CS846), procollagen type 2 C-propeptide (CPII) and glycosaminoglycans (GAG) appeared not modulated. Our data suggest that physiological compression of OA human cartilage modulates the inflammatory milieu by differently affecting the expression of components and homeostasis regulators of the cartilage extracellular matrix.

## Introduction

Articular cartilage is a connective tissue formed by an extracellular matrix (ECM) rich in collagen 2 and proteoglycans and by a single cell type, the chondrocyte, responsible for the synthesis and the maintenance of the ECM integrity [1]. Cartilage protects the surface of the bones in the diarthroidal joints reducing friction and distributing the load. Anabolic pathways induce the production of ECM components, mainly collagen 2 and proteoglycans; on the contrary, catabolic pathways promote the increase of ECM degrading enzymes, such as Matrix Metalloproteinase 13 (MMP-13) and a disintegrin and metalloproteinase with thrombospondin motifs (ADAMTS). These pathways are controlled by the cytokine network, both inflammatory (interleukin [IL]-1β; Tumor Necrosis Factor α [TNFα], IL-6, IL-15, IL-17, and IL-18) and anti-inflammatory (IL-4, IL-10, and IL-13) [2].

Mechanical loading is an important factor affecting regulation, development and long-term maintenance of cartilage homeostasis. Chondrocyte mechanotransduction is initiated at the interface between the cell membrane and the ECM [3, 4]; different strains applied on the chondron (defined as the whole of the chondrocyte and the pericellular matrix) in different cartilage zones produce different biological responses [5, 6]. Magnitude, duration and type of mechanical stimuli, activating different mechanosensitive structures, could initiate anabolic or catabolic pathways [7–9]. Accumulating evidence suggests that cartilage loading in the physiological range contributes to cartilage integrity preservation, whereas either reduced or overloading have catabolic effects [10, 11]. Indeed, prolonged exposure to excessive mechanical stress for different reasons, such as obesity [12], occupational activity [13], or agonistic sport activity [14, 15] might favor early OA onset. Excessive mechanical stimulation is involved in the pathogenesis of osteoarthritis. On the other hand, different studies demonstrate that specific mechanical loads can mitigate joint cartilage erosion. Depending on magnitude and duration of the applied load, compressive stimulation can enhance matrix-protein biosynthesis and remodeling, improve mechanical properties and influence cartilage integrity [9, 16–18].

The regulatory mechanisms of joint homeostasis by mechanical stimuli are still not completely elucidated. One of the principal limiting factors is the available cartilage tissue. Actually, different studies have been performed on cartilage from big size animal models, where the amount of tissue is plentiful. Conversely, in humans few data are available on whole cartilage tissue and studies on mechanical stimulation have been conducted mainly on chondrocytes seeded in scaffolds [19–23]. The few studies carried out up to now point at the need for evaluations of full-thickness human cartilage, challenged by the low tissue availability of surgical specimen from knee OA, where cartilage is frequently very scarce. Data obtained by direct *ex vivo* mechanical stimulation of human cartilage explants constitute an interesting source of precious information, since conditions are more similar to the *in vivo* situation, thus allowing to better reproduce the behavior of the chondrocytes in their physiological micromechanical environment.

For this reason, we studied the effects of mechanical loading on full-thickness human cartilage explants. We evaluated the response of human OA cartilage to *ex vivo* physiological compression alone or in the presence of pro- and anti-inflammatory stimuli. The experimental design allowed us to perform histological, immunohistochemical and molecular analyses on several pivotal markers of cartilage structure and metabolism in each single cartilage donor sample, by comparing different stimulation conditions and in the presence/absence of compression.

Obtained data suggest that physiological compression of human OA cartilage is able to influence the inflammatory milieu by modulating the expression of some molecules involved in cartilage matrix metabolism.

## Materials and methods

### Sample collection and experimental design

**Fig 1:**
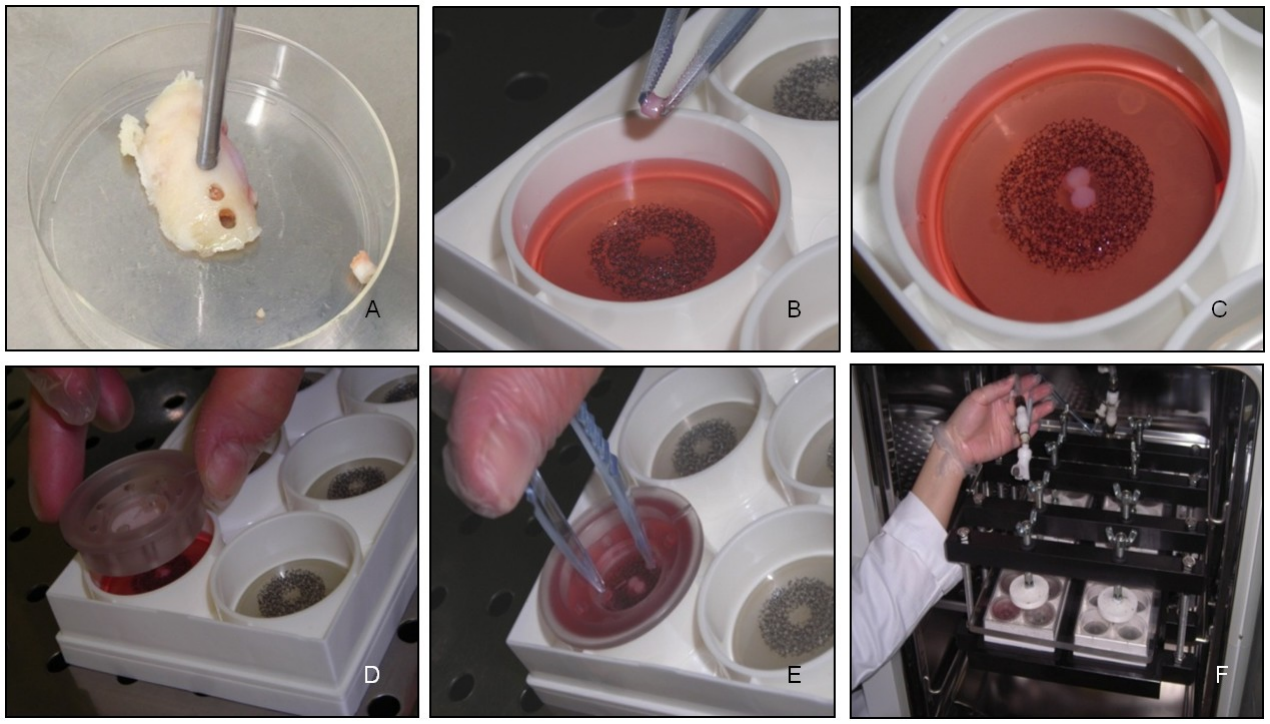
Ex vivo compression experiment set up. (A) Cartilage cylinders were obtained from femoral condyles by tissue coring; (B), (C) cartilage cylinder positioning into the compression plate well; (D), (E) positioning of the stationary platen on the top of the well; (F) assembly of the Flexcell stage presser apparatus in the CO_2_ incubator.

Femoral condyles were collected at time of knee replacement surgery from 10 OA patients (6 men, 4 women, mean age ± standard deviation, SD: 72±7.02 years). Written informed consent was obtained from all patients and the study was approved by the Istituto Ortopedico Rizzoli Ethic Committee (Prot. gen. 0005860). Cylinders of osteo-articular tissue were harvested with a 8 gauge-diameter corer (2.5 mm internal diameter) and subchondral bone was trimmed out. Cartilage explants were cultured overnight in D-MEM (SIGMA, Sigma Aldrich, St. Louis, USA) with 10% heat inactivated FCS (GIBCO, Thermo Fisher Scientific, NY, USA), then serum starved for 24hrs. *Ex vivo* mechanical compression was applied with the FlexCell FX-4000C stage presser apparatus, a computer-regulated bioreactor for compression of tissue samples simulating biological conditions (Flexcell International Corporation, USA). Cartilage cylinders were weighed and positioned into compression plate wells (consisting of a chamber bonded to the bottom of a flexible silicon rubber membrane) with 2ml D-MEM without FCS. The cylinders were carefully oriented and all placed in the same position with respect to the mechanical stimulation, with the upper layer of the cartilage facing up. A stationary platen was added to each well and screwed down until it touched the top of the tissue, following manufacturer’s instructions. Air pressure applied to the bottom caused the chamber to rise and apply an unconfined compression to the cartilage explants. Fig 1 displays positioning of culture explants in the stage presser apparatus of the Flexcell bioreactor. All the experiments were performed at 37°C, 5% CO_2_. Intermittent controlled physiological compression with a sinusoidal waveform was applied at 1Hz frequency for three rounds of 4hrs with 20 hrs interval, 6% compression (36 kPa). Compression conditions were chosen based on literature data [24] and on preliminary experiments (not shown) in order to apply a regime in the physiological range of intensity. Paired control explants were maintained at 37°C, 5% CO_2_ in unloaded conditions. For each donor, three conditions were tested: basal; stimulated with 2ng/ml IL-1β (pro-inflammatory stimulus; rhIL-1β, R&D Systems, Minneapolis, USA); and stimulated with 10ng/ml IL-4 (selected for its pivotal role in chondrocyte anabolic response to mechanical stimulation; rhIL-4, R&D Systems, Minneapolis, USA). For each experimental condition at least two 2.5 mm diameter explants (depending on the cartilage available from donor condyles) were used. After compression, cartilage samples and culture supernatants were immediately recovered for downstream analyses of cartilage homeostasis markers. Half tissue samples (at least one explant) were snap frozen and included in Optimal Cutting Temperature compound (OCT) for histology and immunohistochemistry and stored at -80°C. The remaining samples (at least one explant), as well as supernatants, were frozen (-80°C) for molecular analyses and soluble factor determination, respectively.

### Histology

Histology of cartilage samples was done by conventional Safranin-O staining. Briefly, serial sections of OCT-included explants, 5 μm thick, were cut using a Cryostat Leica CM-1950, thawed, fixed in 4% paraformaldehyde at room temperature for 30 minutes, rehydrated and stained with Haematoxylin/Eosin, 0.1% Safranin-O and 0.02% Fast Green (SIGMA-ALDRICH, Munich, Germany) to assess general cartilage morphology, matrix proteoglycan content and to perform the histopathology grading score. Representative slices for each donor and each culture condition (trasversal sections in the central region of the entire explants) were evaluated. The grading was determined independently by three biologists with experience in cartilage histopathology using the OARSI criteria [25]. All the evaluations were performed with Eclipse 90i microscope and NIS elements software (NIKON, NY, USA).

### Gene expression analysis

One/two cartilage cylinders (depending on the available material), both compressed and uncompressed and ranging from 20 to 80 mg weight were used for gene expression analysis. Liquid nitrogen frozen samples were pulverized using the Mikro-Dismembrator S (Sartorius Stedim Italy SpA, Italy) grinding mill in 5 ml PFTE shaking flasks with a stainless steel grinding ball at a shaking frequency of 2000/min for 1 min. Pulverized explants were solubilised in RNA pure isolation reagent (EUROCLONE, Milan, Italy) to extract total cellular RNA and reverse transcription was performed by random hexamer priming using the SuperScript VILO cDNA Synthesis kit (Life Technologies, NY, USA), following manufacturer’s instructions.

Collagen 2 (COL2A1), SOX9, aggrecan, and ADAMTS4 mRNA expression was evaluated by semi-quantitative Real-Time RT-PCR in a Light Cycler Instrument (ROCHE Molecular Biochemicals, Mannheim, Germany) using the SYBR Premix Ex Taq (TAKARA Biomedicals; Tokyo, Japan) with the following protocol: 95°C for 10 sec, followed by 45 cycles at 95°C for 5 sec and 60°C for 20 sec. Primers and PCR conditions for COL2A1, SOX9 and Aggrecan were as described [26]; commercial primers were used for ADAMTS4 expression (Qiagen, Hilden, Germany; Cat n. PPH14490A).

The C_t_ (Cycle threshold) values were determined for each sample. Amplicon specificity was checked with gel electrophoresis and confirmed at each run by melting curve analysis. Messenger RNA levels were quantified in respect to the glyceraldehyde-3-phosphate dehydrogenase (GAPDH) housekeeping gene following the formula (1+E)^ΔC^_T_, where E represents the reaction efficiency (approximated to 1 because >90% for all the transcripts) and ΔC_T_ the difference between the GAPDH and the specific crossing point for each sample.

### Immunohistochemistry

Immunohistochemistry analysis was carried out to identify SOX9 and IL4-Rα positive cells within cartilage. Paraformaldehyde-fixed 5 μm thick sections were rehydrated and incubated overnight at 4°C with primary antibodies against SOX9 (1 μg/ml, MAB5535, Millipore, Massachusetts, USA) and against IL-4Rα (10 μg/ml, MAB230, R&D System, Minneapolis, USA). Binding was developed for 5–15 minutes with a biotin/streptavidin amplified, alkaline phosphatase-based detection system and with fuchsin as a substrate (BIOGENEX, San Ramon, USA). After nuclear counterstaining with hematoxylin for 1 minute, sections were mounted in glycerol gel and stored at 4°C for subsequent analysis. Negative control samples were processed according to the above-described procedure, omitting the primary antibody. Specificity controls were assessed using isotype control antibodies (R&D Systems, Minneapolis, USA) at the same concentration of the corresponding primary antibody. Quantification was performed on representative slices for each donor and each condition, spanning the tissue from the superficial to the deep zone. Results of the immunohystochemistry analysis were expressed as percentage of positive chondrocytes for each marker over the total number of cells. All the samples were analyzed with Eclipse 90i microscope and NIS elements software (NIKON, NY, USA).

### Soluble factor quantification

The level of soluble factors in the supernatants of cartilage explants was measured by ELISA tests, according to manufacturer’s instructions. COMP (Cartilage Oligomeric Matrix Protein), VEGF (Vascular Endothelial Growth Factor) and MMP-13 (Matrix Metalloproteinase 13) commercial ELISA kits were obtained by R&D System (Minneapolis, USA); ADAMTS4 and ADAMTS5 by Mybiosource (San Diego, USA); C2C (Neoepitope generated through cleavage of type-II Collagen by collagenases) and CS-846 (Chondroitin Sulfate 846 Epitope) by Ibex (San Jose, USA), and PIICP (Collagen synthesis C-terminal Propeptide of Collagen Type-II) by Life Science Inc. (Chestertown, USA).

The amount of Glycosaminoglycan (GAG) release in culture supernatants was determined by a spectrophotometric Dimethylmethylene blue (DMMB) assay (Sigma, St. Louis, USA) [27].

All results were normalized to tissue weight of the corresponding cartilage explants.

### Statistical analysis

Data are presented as medians, interquartile ranges, minimum and maximum values; percentages, means± standard deviations, as appropriate. Differences among culture conditions were analyzed using the Friedman-ANOVA test. Differences within groups were analyzed by the Wilcoxon matched pairs test followed by Bonferroni’s correction for multiple comparisons. Differences between compressed and uncompressed samples in the same culture conditions were analyzed by the Wilcoxon matched pairs test.

The level of statistical significance was set at p<0.05 (a value of p<0.017 was considered significant after Bonferroni’s correction). Data were analyzed using the Statistica 7 software (StatSoft. Inc., Tulsa, USA).

## Results

### Histology

**Fig 2:**
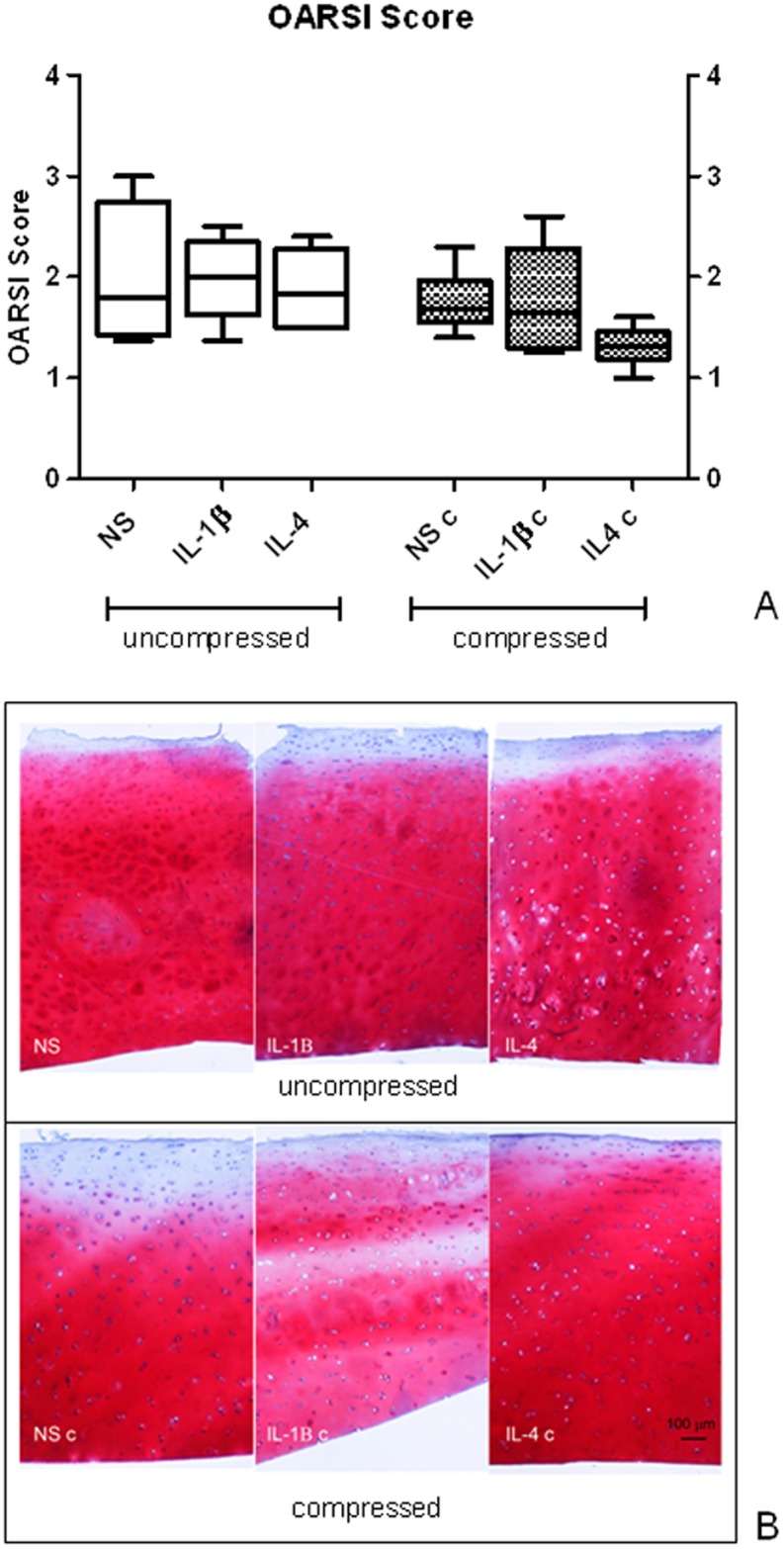
Knee OA explant histological score. A) OARSI histological score evaluation performed on uncompressed (left panel) and compressed (right panel) human OA explants. Bars indicate medians, boxes 25% to 75% percentiles, whiskers Min to Max values; B) representative images of Safranin-O/Fast Green staining of uncompressed (upper panels) and compressed (lower panels) OA cartilage samples.

To assess cartilage degeneration level, the OARSI histological score evaluation was performed on biopsies obtained from the different donors. Mean histological score in basal conditions was 2.00 ± 0.67 (mean ± SD). To exclude a sampling bias in cartilage explant distribution to different experimental conditions, the OARSI score was also evaluated on NS, IL-1β-treated and IL-4-treated samples, both compressed and uncompressed (Fig 2). A borderline significance was observed by comparing all experimental conditions (Friedman ANOVA test, p = 0.044). This not completely homogeneous distribution was not attributable to specific experimental groups even if, as evidenced in Fig 2A, IL-4-treated samples exposed to compression showed a definitely lower histological score than other groups, despite this fails to reach statistical significance. This suggests a synergistic positive effect of compression combined with IL-4.

### Gene expression

**Fig 3:**
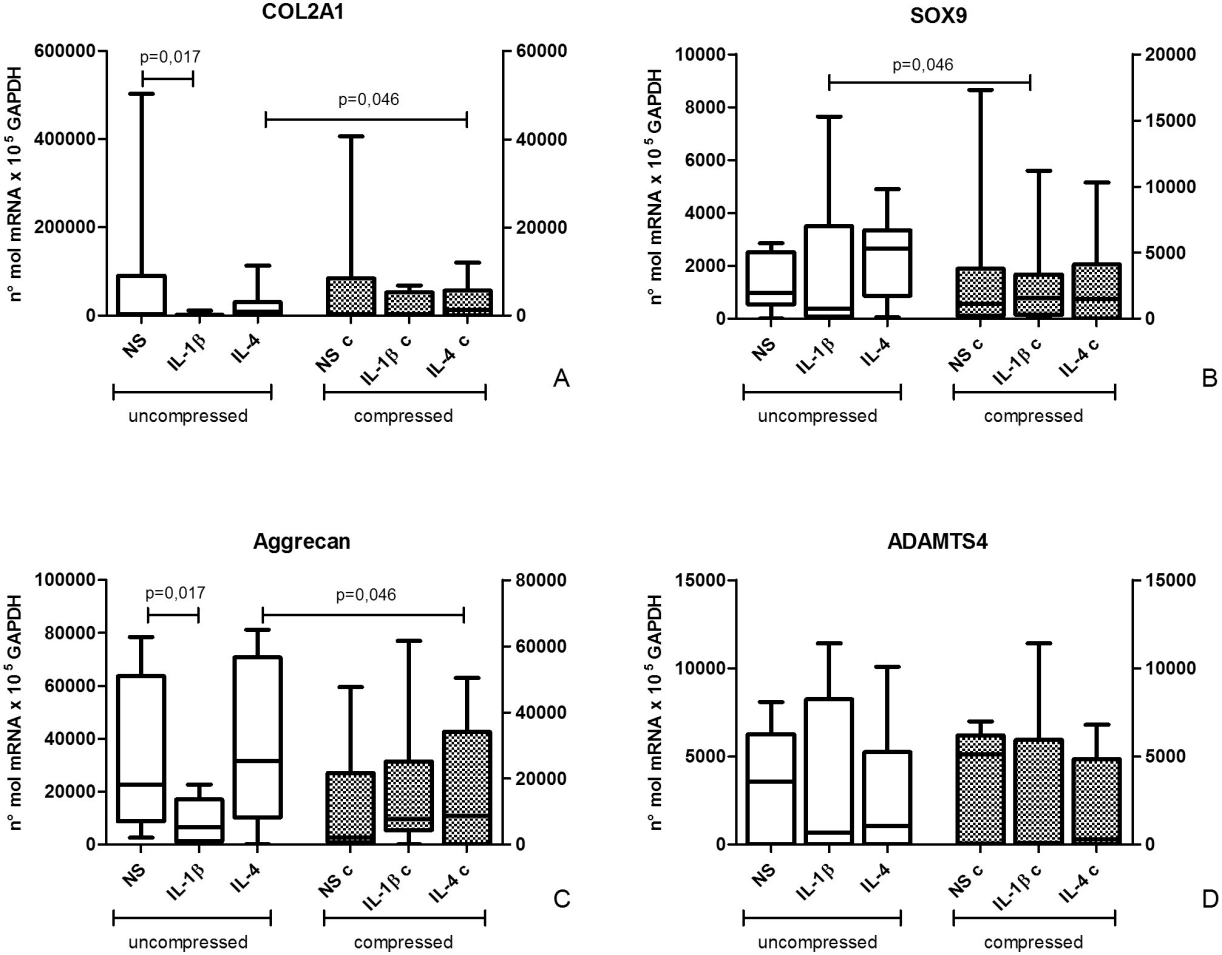
Effect of compression on cartilage marker mRNA expression. COL2A1 (A), SOX9 (B), Aggrecan (C) and ADAMTS4 (D) mRNA expression in uncompressed (left) and compressed (right) human OA cartilage explants by semi-quantitative real time RT PCR. Bars indicate medians, boxes 25% to 75% percentiles, whiskers Min to Max values.

The amount of cartilage recovered from OA donor samples is low. Despite this limitation, biopsy pulverization in liquid nitrogen followed by RNA extraction allowed to obtain a sufficient total RNA amount to analyze different cartilage markers in all experimental conditions: COL2A1, SOX9, Aggrecan and ADAMTS4 (Fig 3).

In general, after compression, collagen 2 expression levels appeared lower than in uncompressed ones, even if this was statistically significant only for IL-4 treated samples (p = 0.046). COL2A1 mRNA expression in uncompressed samples was different among culture conditions (Friedman ANOVA test, p = 0.015). In particular, a COL2A1 decrease was observed after IL-1β incubation compared with unstimulated samples (p = 0.017). This decrease was not detectable in the corresponding compressed samples, where COL2A1 mRNA expression was not different among the three experimental conditions (NS, IL-1β and IL-4). This could suggest that compression is able to counteract the decreased COL2A1 expression induced by IL-1β.

SOX9 mRNA expression did not show significant modifications after cytokine treatment, both in compressed and uncompressed samples. By comparing compressed and uncompressed groups, a mild increase in SOX9 expression after compression in IL-1β-treated samples was observed (p = 0.046). Again, this could suggest a counteracting effect of compression on IL-1β activity.

Aggrecan mRNA showed lower expression levels in uncompressed samples stimulated with IL-1β than in unstimulated (p = 0.017), similarly to collagen 2 mRNA behavior. By comparing compressed and uncompressed groups, a negative trend was observed in aggrecan expression after compression, except for IL-1β treated samples, where Aggrecan expression was increased (p = 0.035), suggesting once again a counteracting effect of compression on IL-1β effect.

In addition, samples incubated with IL-4 showed a difference in Aggrecan mRNA expression between uncompressed and compressed conditions (p = 0.035).

As concerning ADAMTS molecules, ADAMTS4 expression was evaluated at mRNA level since this molecule was shown to be correlated to the degree of cartilage destruction and to be IL-1β-inducible [28]. No differences in ADAMTS4 mRNA expression were highlighted among all tested experimental conditions.

### Immunohistochemistry

**Fig 4:**
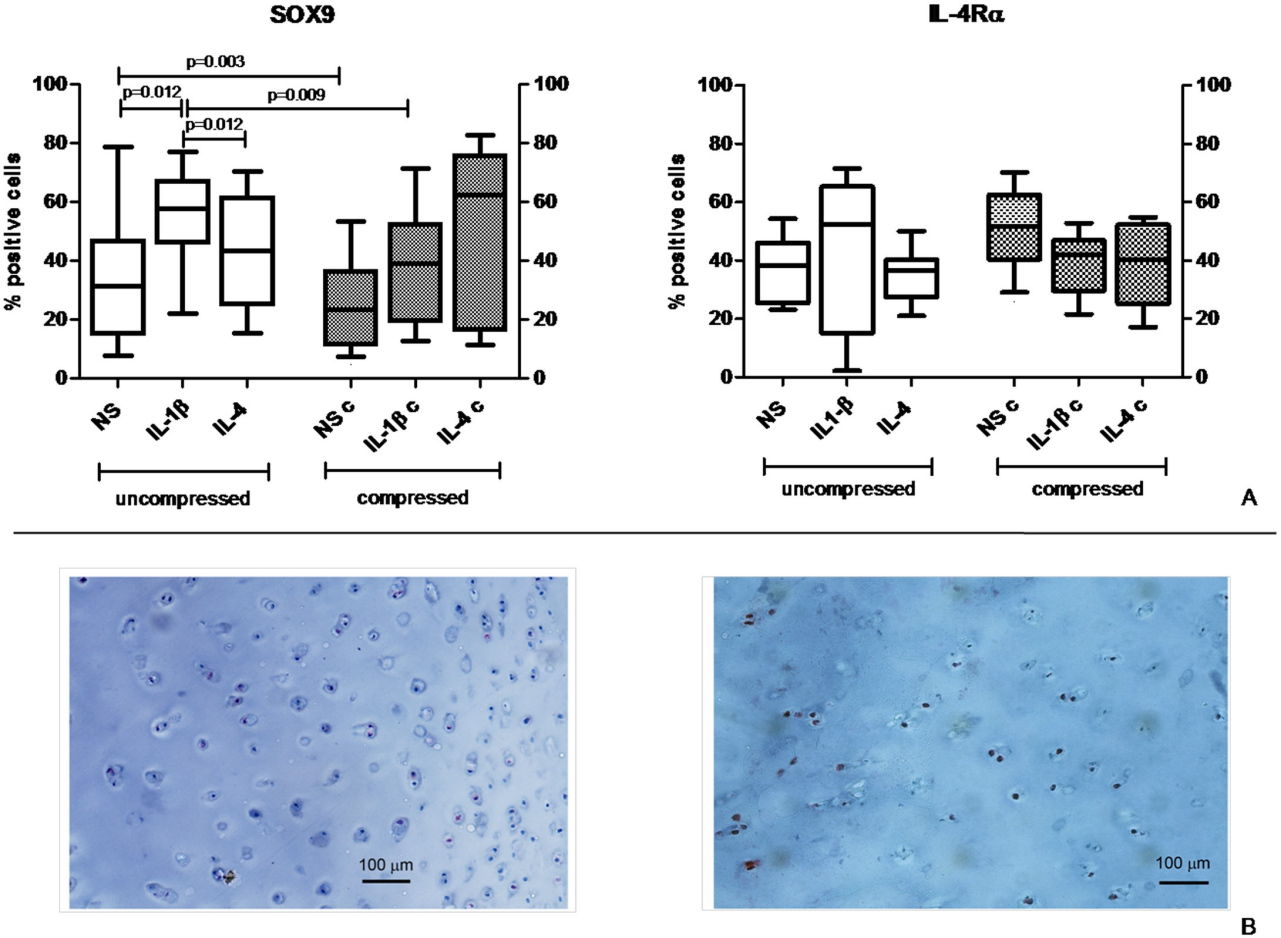
Effect of compression on cartilage marker protein expression. A) Immunohistochemistry analysis of SOX9 and IL-4Rα. Percentage of positive cells on total analyzed cells in uncompressed (left panel) and compressed (right panel) human OA cartilage explants is represented. Bars indicate medians, boxes 25% to 75% percentiles, whiskers Min to Max values; B) representative images of SOX9 (left) and IL-4Rα (right) staining of OA cartilage samples.

Histological sections were tested for SOX9 and IL-4Rα proteins (Fig 4).

The percentage of SOX9 positive chondrocytes showed a decrease in the compressed culture in both unstimulated and IL-1β stimulated conditions when compared to the corresponding uncompressed conditions (p = 0.003 and p = 0.009, respectively). IL-1β treatment also significantly increased the percentage of SOX9 positive condrocytes in uncompressed condition, whereas no increase was found in compressed cultures. Cultures stimulated with IL-4 presented a higher percentage of SOX9 positive chondrocytes (30% on average) in compressed conditions when compared to paired uncompressed conditions, even if not reaching statistical significance.

The percentage of IL-4Rα positive chondrocytes was not significantly modified by compression or culture conditions (with/without IL-1β or IL-4).

### Soluble factor quantification

#### Cartilage matrix anabolic/catabolic factors

**Fig 5:**
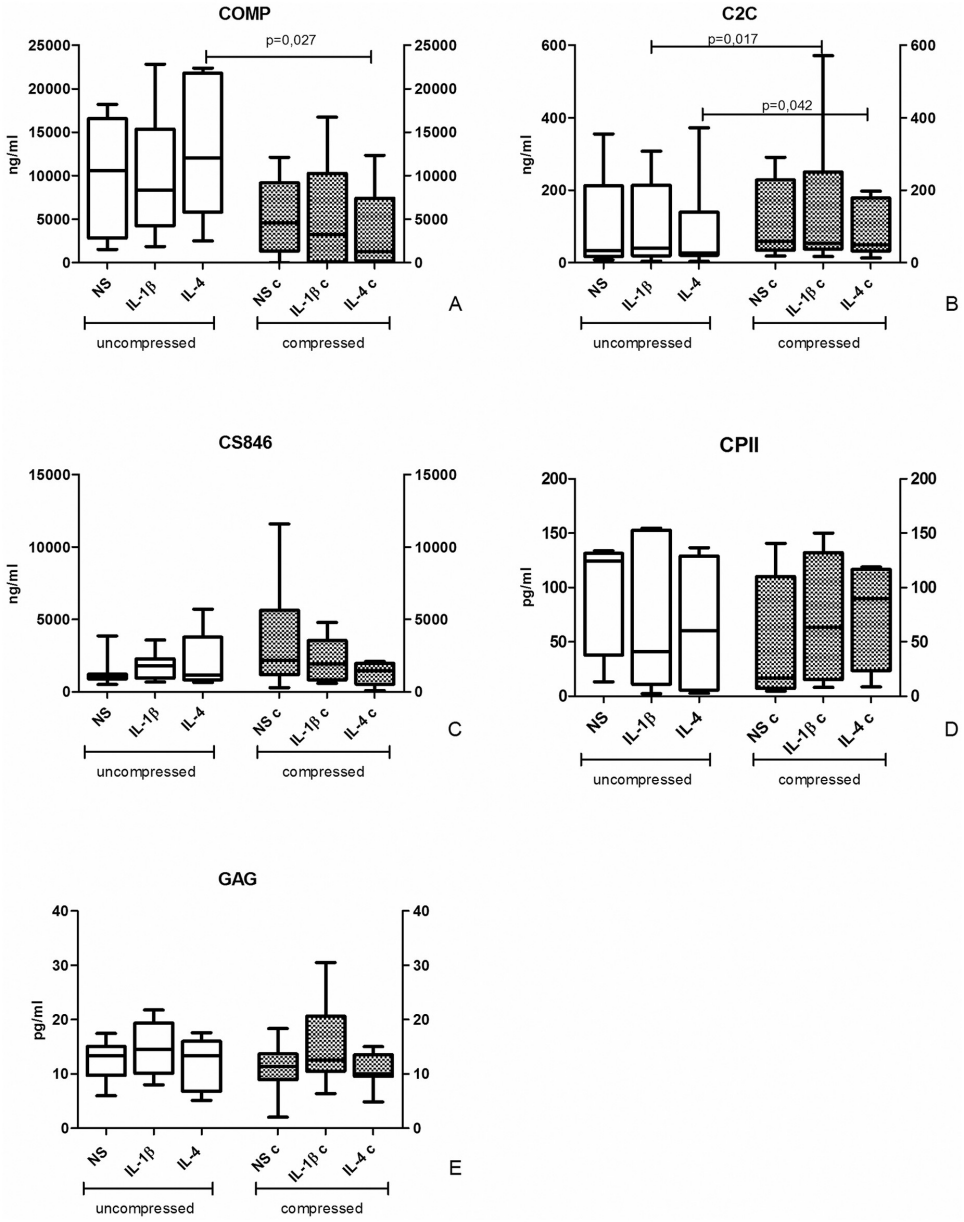
Effect of compression on cartilage matrix anabolic/catabolic factor release. ECM components in culture supernatants of uncompressed (left) and compressed (right) human OA cartilage explant cultures. Protein levels were determined by ELISA tests. Bars indicate medians, boxes 25% to 75% percentiles, whiskers Min to Max values.

Supernatant levels of collagen catabolic molecules (Fig 5a and 5b) COMP (marker of matrix degradation) and C2C (marker of collagen 2 degradation) were similar among different stimulation conditions within uncompressed or compressed groups. After compression, a significant down-modulation of COMP was observed in IL-4-trated samples (p = 0.027). As concerning C2C, the opposite behavior was observed for IL-4 (p = 0.042) together with C2C up modulation after compression in IL-1β-treated samples (p = 0.017).

CS846 (marker of aggrecan turnover) and CPII (marker of type II procollagen synthesis) anabolic factor amount was not significantly influenced by culture conditions and compression (Fig 5c and 5d), even if a trend in up-regulation of CS846 after compression in unstimulated samples was observed.

Similarly, GAG release was not modified by culture conditions and loading (Fig 5e).

### ECM degrading enzymes

**Fig 6:**
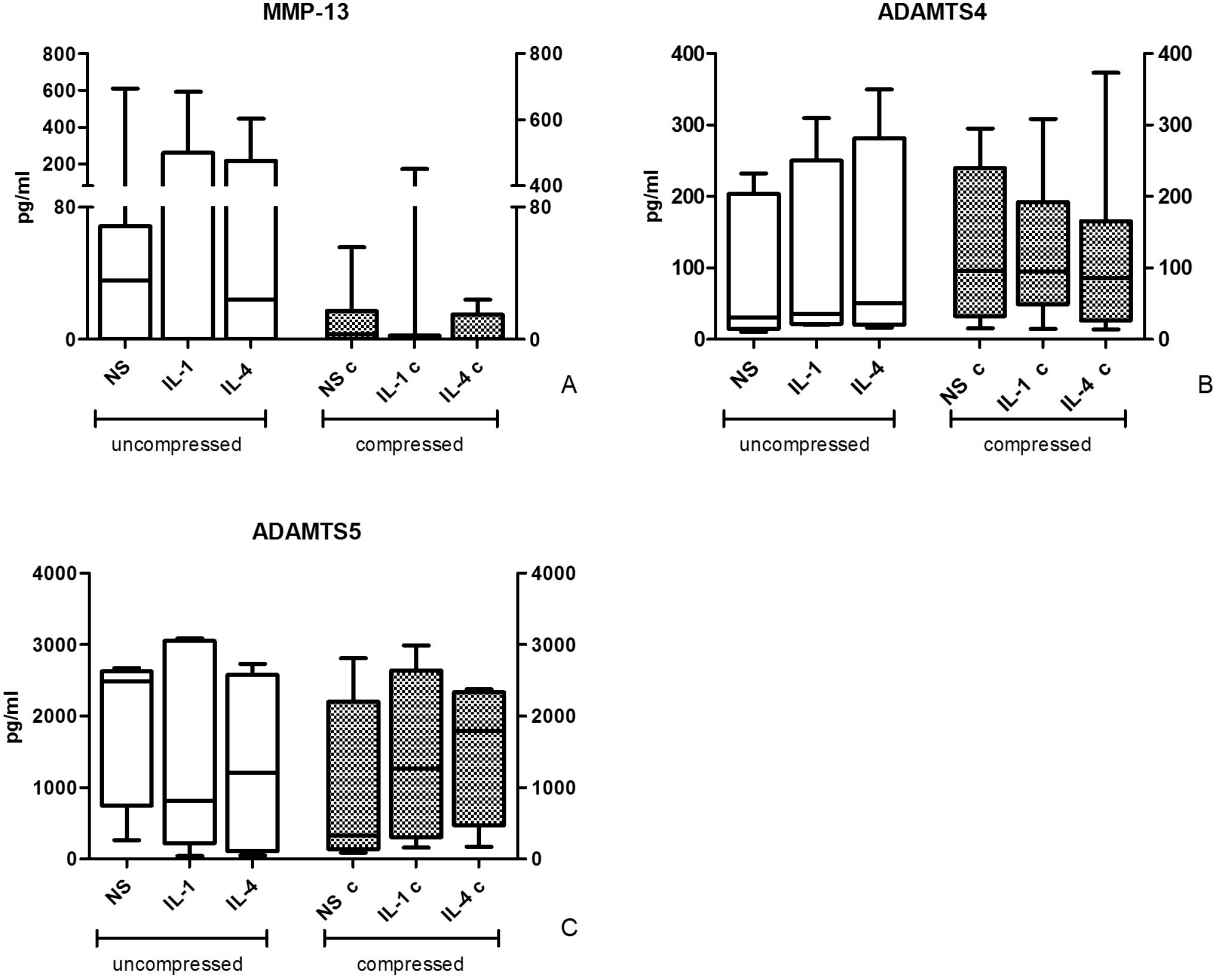
Effect of compression on ECM degrading enzyme release. Degrading enzyme levels in culture supernatants of uncompressed (left) and compressed (right) human OA cartilage explant cultures. Protein levels were determined by ELISA tests. Bars indicate medians, boxes 25% to 75% percentiles, whiskers Min to Max values.

As to catabolic enzymes is concerned, no significant differences for MMP-13 were observed (Fig 6a). Again, a trend suggesting a reduction of MMP-13 in culture supernatants after compression can be observed, even if not reaching statistical significance. In particular, maximum MMP-13 level in compressed conditions was on average more than 3 fold lower than the corresponding one in uncompressed conditions (up to 20 fold lower following IL-4 stimulation).

ADAMTS4 and ADAMTS5 (Fig 6b and 6c) levels in supernatants of compressed and uncompressed conditions did not differ. Compression resulted in a border line increase (p = 0.049) in ADAMTS4 concentrations in IL-4-treated samples compared to uncompressed samples.

### VEGF

**Fig 7:**
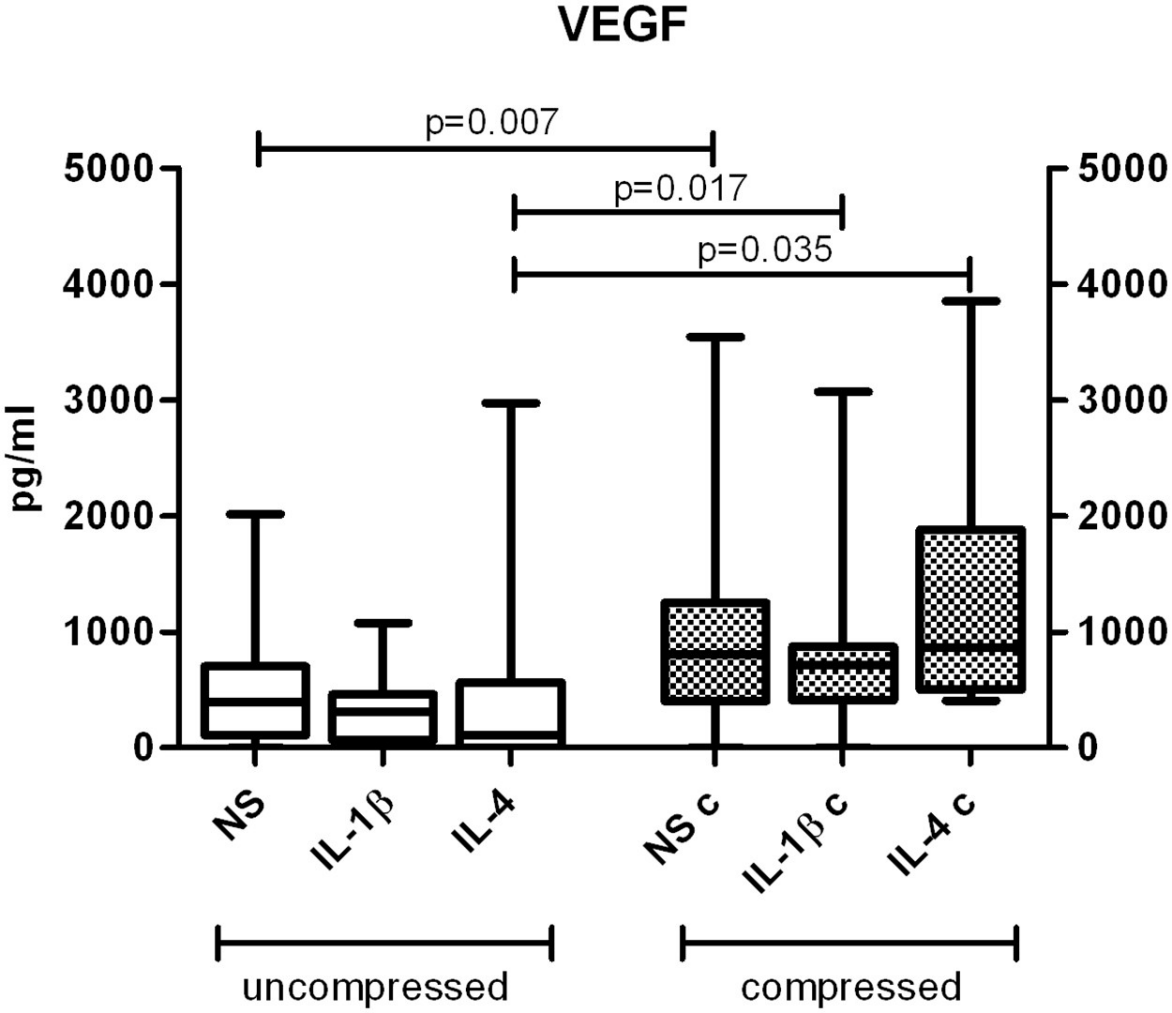
Effect of compression on VEGF release. VEGF levels in culture supernatants of uncompressed (left) and compressed (right) human OA cartilage explant cultures. Protein levels were determined by ELISA tests. Bars indicate medians, boxes 25% to 75% percentiles, whiskers Min to Max values.

VEGF levels in culture supernatants appeared up-regulated by compression (Fig 7) both in untreated and interleukin-treated samples (NS p = 0.007; IL-1β p = 0.017 and IL-4 p = 0.035).

## Discussion

Mechanical stimulation appears to play a key role in cartilage homeostasis maintenance, but it can also contribute to OA pathogenesis, depending on the intensity and frequency of the exerted load.

This study aimed at setting up an experimental model of *ex vivo* mechanical loading of human articular OA cartilage tissue at physiological intensity. Its added value lies in the use of explanted human tissues and in the execution of compression regimes on full-thickness knee OA cartilage cylinders, in which all the layers of cartilage are represented in an *in vivo*-like environment. Since available data on human cartilage mechanobiology are mostly obtained on isolated chondrocytes in monolayers or seeded in scaffolds [19–23], it appears interesting to evaluate human tissue response in conditions mimicking the real tissue environment. We tested a series of anabolic and catabolic cartilage markers at mRNA and protein level to investigate if physiological mechanical stimulation could have a beneficial effect favoring anabolic pathways and counteracting catabolic ones. The scarce available tissue in femoral condyles from OA patients undergoing knee replacement makes it challenging to set up different experimental conditions and to perform different analyses in a single sample. We found a wide inter-individual variability despite a quite homogeneous macroscopic and microscopic (OARSI score) level of cartilage degeneration. The observed variability may have masked some effects of compression and cytokine treatments. Actually, in several cases we observed increasing or decreasing trends in the expression of some molecules suggesting a positive effect of compression, but failing in reaching statistical significance. The advanced OA chondrocyte derangement could have affected cellular ability to respond or have modified the intensity of the response to compression itself.

The OARSI score, performed to assess the general cartilage status of degeneration and to exclude sampling bias in attributing tissue cylinders to the different experimental conditions, indicated that the level of cartilage degeneration did not significantly change among the different applied stimuli. However, even in the absence of a statistical significance samples treated with IL-4 in combination with compression showed the best histological score in all analyzed samples, suggesting a positive and synergistic effect of physiological loading combined with IL-4 on cartilage status, in accordance to literature data. This is in agreement with the described anabolic activity of this cytokine. Actually, the IL-4/IL-4 receptor system is fundamental for the ability of chondrocytes to respond to mechanical stimuli. In physiological conditions mechanical stimulation induces a local production of IL-4 activating a chondroprotective autocrine/paracrine signal that stimulate matrix synthesis and inhibits degradation [28].

In general, the results obtained by comparing compressed to uncompressed samples at mRNA and protein level are suggestive of a partial response with some molecules displaying expression regulation and other without significant modifications.

The modulation of a set of markers by physiological compression indicates that also tissues of pathological derivation seem to maintain their mechanoreceptor functions. Despite inter-individual variability, IL-1β pro-inflammatory treatment significantly reduced collagen 2 and Aggrecan expression as expected. This effect was no longer observed after compression, suggesting that physiological loading can counteract the pro-inflammatory milieu. Conversely, no clear effect was highlighted in SOX9 and ADAMTS4 expression. Actually, in isolated human chondrocytes seeded in 3D scaffolds an increase in SOX9 mRNA expression was observed, but after application of higher intensity loads [29] (25% vs 6% used in our study). Conversely, Wang et al. (2009) found that compression had no effect on SOX9 expression in bovine chondrocytes [23]. No consensus was therefore obtained on the effect of loading on SOX9 expression.

Our phenotype analysis was only partly in agreement with gene expression data. SOX9 immunohistochemistry data showed lower percentages of positive cells after compression in NS and IL-1β. Conversely, IL-4 combined with compression induced a trend towards SOX9 upregulation, in keeping with IL-4 known role in the mechanotransduction pathway and again suggesting the synergistic activity of IL-4 with mechanical stimulation.

The lack of modulation on IL-4Rα protein expression confirms the results of our previous paper in which IL-4R was not modulated by different stimuli [28].

Soluble factor analysis showed some modulation of cartilage turnover markers. This was evident for COMP, the presence of which is involved in the assembly of the collagen fibrils and in the stabilization of the ECM and seems to have a fundamental role in the bearing of the mechanical load [30]. Its appearance indicates ECM degradation, therefore, the general reduction in COMP levels in compressed samples and in particular in samples where compression was combined with IL-4 suggests that compression promotes the slowdown of cartilage degradation and that this effect is synergistic with IL-4.

Opposite to what expected, C2C and CPII did not respond in a specular way despite their opposite tasks, being respectively markers of collagen 2 degradation and of procollagen type 2 synthesis.

As concerning the markers of ECM proteins other than collagen (CS846 and GAG), they seem to be not modulated by either compression or cytokine stimulation. Results about GAG are supported by similar evidences in bovine models [8].

MMP-13, ADAMTS4 and ADAMTS5, pivotal matrix degrading enzymes in OA, responsible for the initial cleavage of collagen 2 and Aggrecan, respectively, appeared not significantly modulated whatever the culture conditions. Anyway, a clear trend of reduced MMP-13 levels in all samples after compression is evident. We cannot exclude that these results were influenced by the inter-individual variability among samples.

Interestingly, the Vascular Endothelial Growth Factor (VEGF) supernatant levels were higher after compression in all stimulation conditions. This confirms previous observations from our laboratory in which VEGF was able to down-modulate chondrocyte activities related to catabolic events involved in OA cartilage degradation, such as caspase-3 and cathepsin B [31]. Furthermore, our data are in line with Beckmann et al. [32], demonstrating that also minor mechanical forces (1% -16% of cyclic equibiaxial strains) stimulated isolated chondrocytes and chondrocyte cell lines to produce VEGF. Even if VEGF is well known to play a master role in angiogenesis and in negative regulation of cartilage growth by stimulating vascular invasion and ossification, these findings could indicate that mechanically solicited chondrocytes activate an early production of this factor, attempting to preserve cartilage integrity [31].

The challenging experimental design including the comparison of several experimental conditions set up from the cartilage explants of each single OA donor was probably responsible for the observed inter-individual heterogeneity. This could partly explain the non homogeneous response to loading of some markers and reflect the lack of homogeneous distribution/presence of the chondrocytes within the different cylinder tissue samples. Actually, compressive loading could induce different responses in different cartilage zones, as reported by Jeon et al. [33]. From this perspective, we are planning to develop our study for a better correlation between cartilage response to compression and cartilage degenerative status. Since the articular surface is not homogeneous, probably we will obtain more information by discriminating different areas in the same sample.

The observed response to loading could also be related to the advanced derangement found in late OA chondrocytes, possibly affecting the cellular response to mechanical stimulation. Samples of our cultures derive from patients with advanced OA, so it is reasonable to assume that chondrocytes in a pathological stress condition are no more able to react to added stimuli by activating a coordinated anabolic response.

Donor age is an additional aspect possibly affecting chondrocyte ability to produce cartilaginous ECM components [34].

Another aspect to be considered is that different molecules can be modulated at different times after compression, both at mRNA and protein level, therefore the moment we collected samples for RNA and protein analysis could not be optimal for all markers. Only kinetic evaluations of each marker, requiring more available tissue, could clarify this point.

Moreover, in patients with OA, a compensatory higher synthesis of collagen 2 and Aggrecan in respect to normal subjects is described and this could mask the increase of these proteins due to mechanical load [35]. Since we had no normal cartilage explants available, we could not establish differences between the tissues of our explants deriving from patients affected by OA and healthy cartilage.

From a rehabilitation perspective, taken together our results suggest a partial efficacy of physical activity on patients with already established arthritic disease. Several studies seem to indicate a beneficial effect of physical activity on the joints of healthy subjects [36]. On the other hand, physical activity effects on OA patients are controversial, even if functional improvement is described, sometimes accompanied by pain reduction, and no OA progression [37].

In conclusion, our data suggest that physiological compression of OA human cartilage tissue influences the effect of the inflammatory milieu by modulating cartilage matrix component metabolism, even if with a complex profile, and this is in line with data from *in vitro* and animal studies. Moreover, a combined positive effect of IL-4 and compression was evidenced. These data stimulate further studies to better elucidate the role of mechanotransduction on cartilage behavior both in normal and pathologic conditions.

## Supporting information

S1 Fig Representative collagen 2 staining of human OA cartilage. (TIF)

S2 Fig Representative aggrecan staining of human OA cartilage. (TIF)

## References

[1] Servin-Vences MR, Moroni M, Lewin GR, Poole K. Direct measurement of TRPV4 and PIEZO1 activity reveals multiple mechanotransduction pathways in chondrocytes. Elife. 2017;6. 10.7554/eLife.21074 PMC5279942 28135189

[2] Wojdasiewicz P, Poniatowski LA, Szukiewicz D. The role of inflammatory and anti-inflammatory cytokines in the pathogenesis of osteoarthritis. Mediators Inflamm. 2014;2014:561459 10.1155/2014/561459 24876674 PMC4021678

[3] Varady NH, Grodzinsky AJ. Osteoarthritis year in review 2015: mechanics. Osteoarthritis Cartilage. 2016;24(1):27–35. 10.1016/j.joca.2015.08.018 26707990 PMC4693146

[4] Wang N, Tytell JD, Ingber DE. Mechanotransduction at a distance: mechanically coupling the extracellular matrix with the nucleus. Nat Rev Mol Cell Biol. 2009;10(1):75–82. 10.1038/nrm2594 19197334

[5] Sanchez-Adams J, Leddy HA, McNulty AL, O’Conor CJ, Guilak F. The mechanobiology of articular cartilage: bearing the burden of osteoarthritis. Curr Rheumatol Rep. 2014;16(10):451 10.1007/s11926-014-0451-6 25182679 PMC4682660

[6] Saxby DJ, Lloyd DG. Osteoarthritis year in review 2016: mechanics. Osteoarthritis Cartilage. 2017;25(2):190–8. 10.1016/j.joca.2016.09.023 28100420

[7] Clements KM, Bee ZC, Crossingham GV, Adams MA, Sharif M. How severe must repetitive loading be to kill chondrocytes in articular cartilage? Osteoarthritis Cartilage. 2001;9(5):499–507. 10.1053/joca.2000.0417 11467899

[8] Nishimuta JF, Levenston ME. Response of cartilage and meniscus tissue explants to in vitro compressive overload. Osteoarthritis Cartilage. 2012;20(5):422–9. 10.1016/j.joca.2012.01.004 22289896 PMC3384701

[9] Sun HB. Mechanical loading, cartilage degradation, and arthritis. Ann N Y Acad Sci. 2010;1211:37–50. 10.1111/j.1749-6632.2010.05808.x 21062294

[10] Leong DJ, Hardin JA, Cobelli NJ, Sun HB. Mechanotransduction and cartilage integrity. Ann N Y Acad Sci. 2011;1240:32–7. 10.1111/j.1749-6632.2011.06301.x 22172037 PMC5007871

[11] Ex vivo mechanical stimulation counteracts IL-1 effect on human oa cartilage explants Assirelli E, Pulsatelli L, Dolzani P, Meliconi R, Facchini A, Neri S. Osteoarthritis and Cartilage, Volume 20, S242—S243

[12] Davis MA, Ettinger WH, Neuhaus JM, Cho SA, Hauck WW. The association of knee injury and obesity with unilateral and bilateral osteoarthritis of the knee. Am J Epidemiol. 1989;130(2):278–88. 10.1093/oxfordjournals.aje.a115334 2750727

[13] Rytter S, Egund N, Jensen LK, Bonde JP. Occupational kneeling and radiographic tibiofemoral and patellofemoral osteoarthritis. J Occup Med Toxicol. 2009;4:19 10.1186/1745-6673-4-19 19594940 PMC2726153

[14] Leung YY, Bin Abd Razak HR, Talaei M, Ang LW, Yuan JM, Koh WP. Duration of physical activity, sitting, sleep and the risk of total knee replacement among Chinese in Singapore, the Singapore Chinese Health Study. PLoS One. 2018;13(9):e0202554 10.1371/journal.pone.0202554 30180156 PMC6122790

[15] Buckwalter JA, Lane NE. Does participation in sports cause osteoarthritis? Iowa Orthop J. 1997;17:80–9. 9234978 PMC2378110

[16] Anderson DE, Johnstone B. Dynamic Mechanical Compression of Chondrocytes for Tissue Engineering: A Critical Review. Front Bioeng Biotechnol. 2017;5:76 10.3389/fbioe.2017.00076 29322043 PMC5732133

[17] Gassner R, Buckley MJ, Georgescu H, Studer R, Stefanovich-Racic M, Piesco NP, et al Cyclic tensile stress exerts antiinflammatory actions on chondrocytes by inhibiting inducible nitric oxide synthase. J Immunol. 1999;163(4):2187–92. 10438960 PMC4967410

[18] Torzilli PA, Bhargava M, Park S, Chen CT. Mechanical load inhibits IL-1 induced matrix degradation in articular cartilage. Osteoarthritis Cartilage. 2010;18(1):97–105. 10.1016/j.joca.2009.07.012 19747586 PMC2818235

[19] Chen CH, Kuo CY, Chen JP. Effect of Cyclic Dynamic Compressive Loading on Chondrocytes and Adipose-Derived Stem Cells Co-Cultured in Highly Elastic Cryogel Scaffolds. Int J Mol Sci. 2018;19(2). 10.3390/ijms19020370 PMC5855592 29373507

[20] Grogan SP, Sovani S, Pauli C, Chen J, Hartmann A, Colwell CW Jr., et al Effects of perfusion and dynamic loading on human neocartilage formation in alginate hydrogels. Tissue Eng Part A. 2012;18(17–18):1784–92. 10.1089/ten.TEA.2011.0506 22536910 PMC3432904

[21] McCutchen CN, Zignego DL, June RK. Metabolic responses induced by compression of chondrocytes in variable-stiffness microenvironments. J Biomech. 2017;64:49–58. 10.1016/j.jbiomech.2017.08.032 28985893 PMC5694357

[22] O’Conor CJ, Leddy HA, Benefield HC, Liedtke WB, Guilak F. TRPV4-mediated mechanotransduction regulates the metabolic response of chondrocytes to dynamic loading. Proc Natl Acad Sci U S A. 2014;111(4):1316–21. 10.1073/pnas.1319569111 24474754 PMC3910592

[23] Wang QG, Magnay JL, Nguyen B, Thomas CR, Zhang Z, El Haj AJ, et al Gene expression profiles of dynamically compressed single chondrocytes and chondrons. Biochem Biophys Res Commun. 2009;379(3):738–42. 10.1016/j.bbrc.2008.12.111 19118531

[24] Goset M, Berenbaum F, Levy A, Pigenet A, Thirion S, Saffar JL, et al Prostaglandin E2 synthesis in cartilage explants under compression: mPGES-1 is a mechanosensitive gene. Arthritis Res Ther. 2006;8(4):R135 10.1186/ar2024 16872525 PMC1779392

[25] Pritzker KP, Gay S, Jimenez SA, Ostergaard K, Pelletier JP, Revell PA, et al Osteoarthritis cartilage histopathology: grading and staging. Osteoarthritis Cartilage. 2006;14(1):13–29. 10.1016/j.joca.2005.07.014 16242352

[26] Neri S, Vannini F, Desando G, Grigolo B, Ruffilli A, Buda R, et al Ankle bipolar fresh osteochondral allograft survivorship and integration: transplanted tissue genetic typing and phenotypic characteristics. J Bone Joint Surg Am. 2013;95(20):1852–60. 10.2106/JBJS.L.01715 24132359

[27] Barbosa I, Garcia S, Barbier-Chassefiere V, Caruelle JP, Martelly I, Papy-Garcia D. Improved and simple micro assay for sulfated glycosaminoglycans quantification in biological extracts and its use in skin and muscle tissue studies. Glycobiology. 2003;13(9):647–53. 10.1093/glycob/cwg082 12773478

[28] Assirelli E, Pulsatelli L, Dolzani P, Platano D, Olivotto E, Filardo G, et al Human osteoarthritic cartilage shows reduced in vivo expression of IL-4, a chondroprotective cytokine that differentially modulates IL-1beta-stimulated production of chemokines and matrix-degrading enzymes in vitro. PLoS One. 2014;9(5):e96925 10.1371/journal.pone.0096925 24819779 PMC4018406

[29] Scholtes S, Kramer E, Weisser M, Roth W, Luginbuhl R, Grossner T, et al Global chondrocyte gene expression after a single anabolic loading period: Time evolution and re-inducibility of mechano-responses. J Cell Physiol. 2018;233(1):699–711. 10.1002/jcp.25933 28369921

[30] Erhart-Hledik JC, Favre J, Asay JL, Smith RL, Giori NJ, Mundermann A, et al A relationship between mechanically-induced changes in serum cartilage oligomeric matrix protein (COMP) and changes in cartilage thickness after 5 years. Osteoarthritis Cartilage. 2012;20(11):1309–15. 10.1016/j.joca.2012.07.018 22868052

[31] Pulsatelli L, Dolzani P, Silvestri T, Frizziero L, Facchini A, Meliconi R. Vascular endothelial growth factor activities on osteoarthritic chondrocytes. Clin Exp Rheumatol. 2005;23(4):487–93. 16095117

[32] Beckmann R, Houben A, Tohidnezhad M, Kweider N, Fragoulis A, Wruck CJ, et al Mechanical forces induce changes in VEGF and VEGFR-1/sFlt-1 expression in human chondrocytes. Int J Mol Sci. 2014;15(9):15456–74. 10.3390/ijms150915456 25257525 PMC4200847

[33] Jeon JE, Schrobback K, Hutmacher DW, Klein TJ. Dynamic compression improves biosynthesis of human zonal chondrocytes from osteoarthritis patients. Osteoarthritis Cartilage. 2012;20(8):906–15. 10.1016/j.joca.2012.04.019 22548797

[34] Jorgensen AEM, Kjaer M, Heinemeier KM. The Effect of Aging and Mechanical Loading on the Metabolism of Articular Cartilage. J Rheumatol. 2017;44(4):410–7. 10.3899/jrheum.160226 28250141

[35] Goldring MB, Marcu KB. Cartilage homeostasis in health and rheumatic diseases. Arthritis Res Ther. 2009;11(3):224 10.1186/ar2592 19519926 PMC2714092

[36] Racunica TL, Teichtahl AJ, Wang Y, Wluka AE, English DR, Giles GG, et al Effect of physical activity on articular knee joint structures in community-based adults. Arthritis Rheum. 2007;57(7):1261–8. 10.1002/art.22990 17907212

[37] Quicke JG, Foster NE, Thomas MJ, Holden MA. Is long-term physical activity safe for older adults with knee pain?: a systematic review. Osteoarthritis Cartilage. 2015;23(9):1445–56. 10.1016/j.joca.2015.05.002 26003947

