# Supplementary figures and images for: Ex vivo physiological compression of human osteoarthritis cartilage modulates cellular and matrix components
Source: PLoS One. 2019 Sep 24;14(9):e0222947. doi: 10.1371/journal.pone.0222947 (PMC6759151; doi:10.1371/journal.pone.0222947)

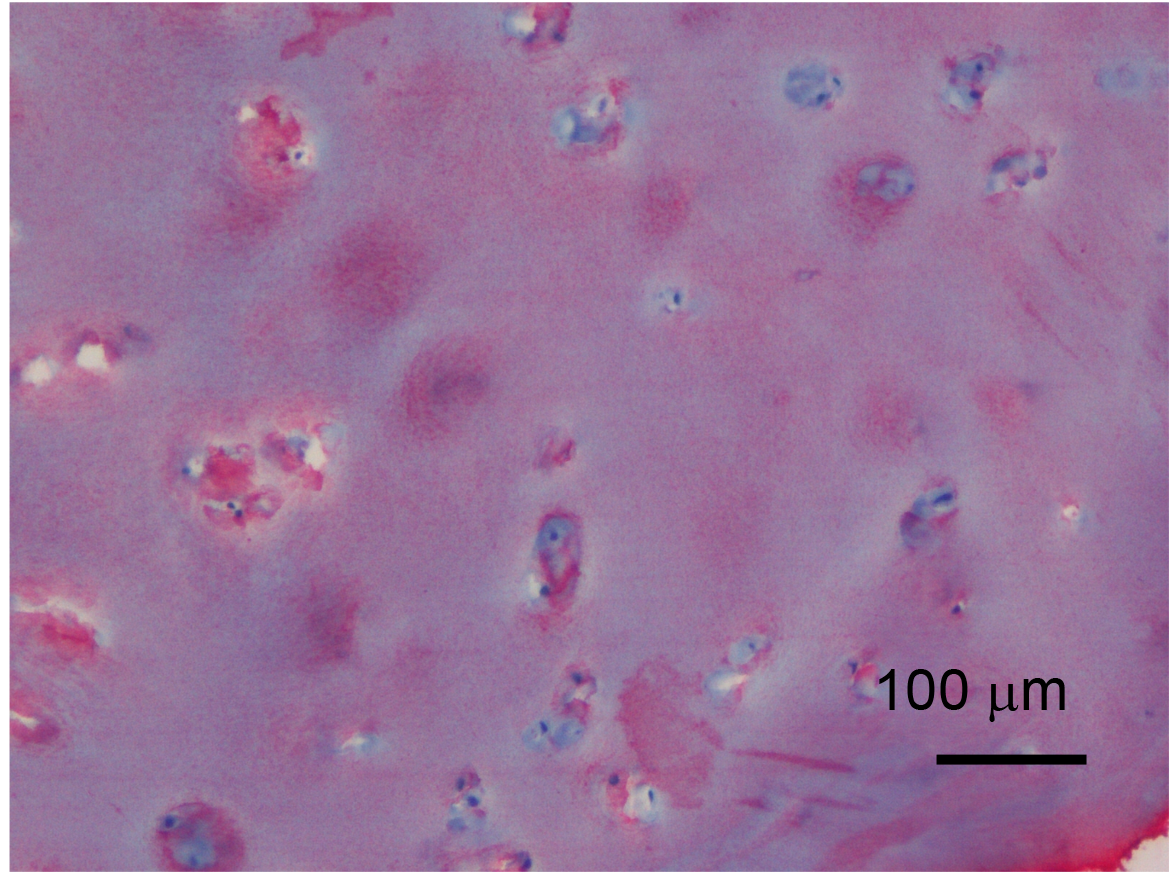

Supplement: S1 Fig — (TIF) [file pone.0222947.s001.tif]

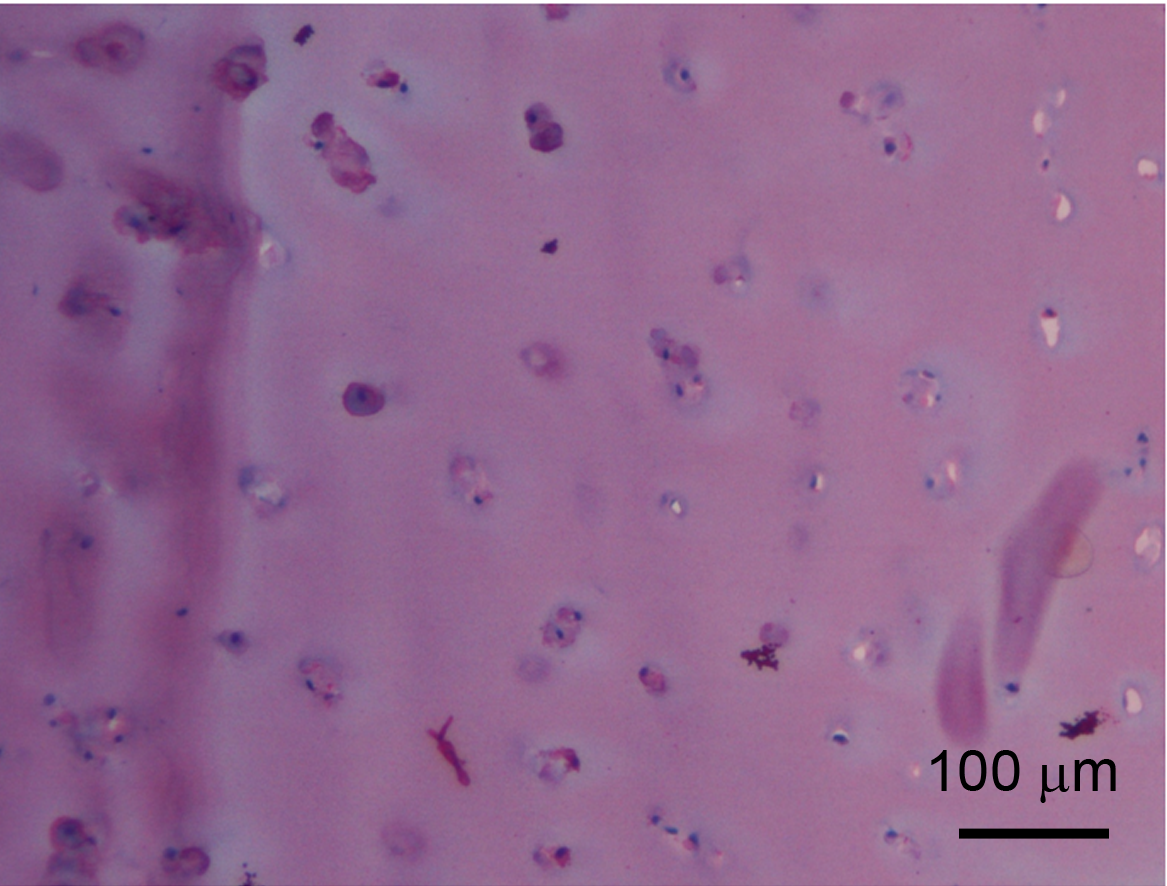

Supplement: S2 Fig — (TIF) [file pone.0222947.s002.tif]
